# Molecular diversity of *Treponema pallidum* subspecies *pallidum* isolates in Amsterdam, the Netherlands

**DOI:** 10.1136/sextrans-2019-054044

**Published:** 2019-08-05

**Authors:** Helene C A Zondag, Akke R Cornelissen, Alje P van Dam, Sylvia M Bruisten

**Affiliations:** 1 Department of Infectious Diseases, Public Health Laboratory, Public Health Service of Amsterdam, Amsterdam, The Netherlands; 2 Department of Medical Microbiology, Amsterdam University Medical Centre, Amsterdam, The Netherlands; 3 Amsterdam Infection & Immunity, Amsterdam University Medical Centre, Amsterdam, The Netherlands

**Keywords:** *treponema pallidum*, molecular typing, macrolide resistance, syphilis

## Abstract

**Objectives:**

The prevalence of syphilis, caused by the spirochete *Treponema pallidum* subsp*. pallidum* (TPA), remains high despite the availability of effective antibiotics. In the Netherlands, most syphilis cases are found among men who have sex with men (MSM). We studied the distribution of TPA strain types by molecular characterisation and related this to available characteristics. In addition, resistance to macrolides was assessed.

**Methods:**

TPA DNA was extracted from 136 genital ulcer swab or skin lesions samples deriving from 135 patients diagnosed with syphilis in 2016 and 2017 at the Public Health Service in Amsterdam, the Netherlands. Molecular typing was done according to the enhanced CDC method (E-CDC), in which three genetic regions of the *arp*, *tpr* and *tp0548* genes are analysed by gel electrophoresis of the *arp* and *tpr* regions and by sequence analysis for the *tp0548* region. Part of the 23S rDNA locus was sequenced to determine the presence of macrolide resistance-associated mutations.

**Results:**

Full E-CDC strain types could be determined for 99/136 (73%) DNA samples, which tested positive in a diagnostic PCR targeting the *polA* gene. Types differed within one patient of whom two samples were available. No association was found between the demographic and clinical characteristics and the TPA types. The most prevalent type was 14d/g, found in 23 of the 99 (23%) fully typed samples. Part of the 23S rDNA locus was successfully sequenced for 93/136 (68%) samples and 83 (88%) contained the A2058G mutation. No A2059G mutation was found.

**Conclusions:**

A broad strain distribution was found. Few subtypes were clonally expanded, and most other subtypes were rare. Detection of the most prevalent strain type, 14d/g, is in concordance with other TPA typing studies. The high prevalence of genetic macrolide resistance indicates that azithromycin is not an alternative treatment option.

## Background

*Treponema pallidum* subsp. *pallidum* (TPA) is the causative pathogen of the venereal disease, syphilis. Men who have sex with men (MSM) are the most important risk group for contracting syphilis. In 2017, MSM accounted for 95,3% of all syphilis cases in the Netherlands.^1^

Molecular epidemiological studies of TPA monitor strain type distribution, and also antimicrobial resistance patterns were studied previously. The genome of TPA is conserved and contains highly polymorphic regions useful for typing. Until now the most commonly used typing method for TPA was developed in 1998^2^ and improved in 2010.^3^ This method, called the enhanced CDC method (E-CDC), is based on the analysis of three distinct genetic regions: the number of repeats in the acidic repeat protein gene (*arp*), the Mse1 restriction pattern of the *Treponema pallidum* repeat protein (*tpr*) genes (*tprE, tprG* and *tprJ*) and sequence analysis of a part of the *tp0548* gene.^3^

The first line antibiotic for syphilis worldwide is penicillin. Alternatives are ceftriaxone, doxycycline and macrolides. Macrolide resistance among TPA has been widely reported, and its prevalence was shown to increase over time.^2^ Two specific mutations, A2058G and A2059G, in the 23S rRNA genes are associated with macrolide resistance.^4^

The objectives of this study were to study the TPA strain distribution and determine the prevalence of macrolide resistance among syphilis cases in Amsterdam, the Netherlands. Furthermore possible associations between demographic and clinical data and strain types were investigated.

## Materials and methods

### Sample selection and testing

Extracted DNA samples were collected from primary ulcer and skin lesion swabs received at the Public Health Laboratory of the Public Health Service (GGD) in Amsterdam for syphilis diagnostics in 2016 and 2017. All tested positive in the routine diagnostic setting based on a qPCR, targeting the *polA* gene.^5^ Serologically, a quantitative rapid plasma reagin (RPR) flocculation test (RPR-Nosticon II; bioMérieux) was performed according to the specifications of the manufacturers.

### Molecular typing

*Arp* and *tpr* regions were amplified using a nested PCR. For the *arp* region, outer primers were used as described,^2^ and inner primers were designed for this study: 5′GCATCTTTGCCGTCCCGTGTGCCT and 5′CGCACGTCCTTTCTGTTCCTCCGGA. The amplified *arp* product was analysed by gel electrophoresis using QIAxcel (Qiagen) or a 2% agarose gel to determine the number of 60 bp repeats. Amplification of the *tprE*, *tprG* and *tprJ* genes were performed as described.^2^ Restriction enzyme *Mse1* digestion pattern of the PCR product was analysed using a QIAxcel. Amplification of the *tp0548* region was performed as described.^3^ The amplified *tp0548* product was sequenced using an ABI3130 (Thermofisher). Sequence analyses were performed using Bionumerics version 7.6.2 (Applied Maths).

### Macrolide resistance

Part of the 23S rDNA locus was amplified using the following primers: 5′GTACCGCAAACCGACACAG and 5′AGTCAAACCGCCCACCTAC. Sanger sequencing was performed to check for the A2058G and A2059G mutations.

### Data analysis

Demographic and clinical data were analysed to investigate associations between typable and non-typable isolates and the presence of macrolide resistance mutations using Fisher’s exact test, considered significant if p<0.05.

## Results

Gender and age data were available for all 135 included patients with a diagnosis of syphilis, contributing 136 samples (from one patient two ulcer swabs were available). The median age was 42 years (IQR: 33–50) and 134/135 (99%) were men. There were 56 samples from 55 patients visiting general practitioners, hospitals or other care providers of whom no additional data were available. The other 80 samples (59%) were collected from patients visiting the STI clinic in Amsterdam. From these clients, sexual orientation based on sexual behaviour in the past 6 months, HIV status and RPR titer were also known. Most were MSM (74/80, 93%) with four men who have sex with men and women (MSMW) (5%) and only two men who have sex with women (MSW) (3%).

More than one-third (29/80, 36%) of syphilis-positive patients attending the STI clinic were HIV positive. For two persons (2%) the HIV status was unknown. RPR titers were classified as low (1:1 to 1:2), middle (1:4 to 1:16) and high (1:32–1:256) and occurred in respectively 20% (16/80), 39% (31/80) and 20% (16/80) in the STI samples. A negative RPR was found for 17/80 (21%) patients.

**Figure 1:**
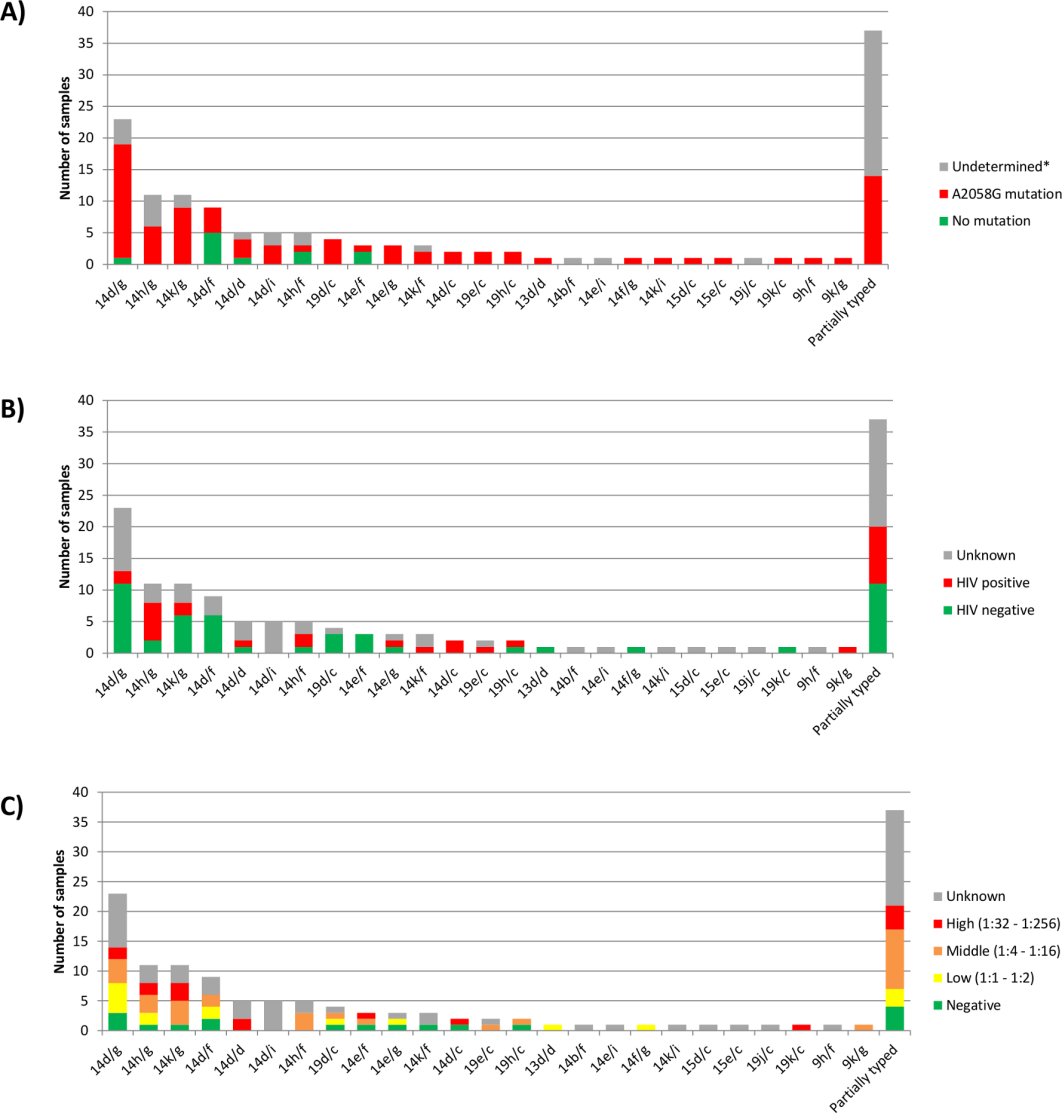
Overview of the 136 samples sorted by E-CDC strain type (A) including presence (green) or absence (red) of the A2058G mutation. The A2059G mutation was not found. (B) E-CDC strain types coloured by HIV status: HIV negative (green), HIV positive (red) and unknown (grey). (C) E-CDC strain types coloured by RPR titer classified as low (1:1 to 1:2, in yellow), middle (1:4 to 1:16, in orange) and high (1:32–1:256, in red) and negative (green). *Part of the 23S rRNA gene was not successfully sequenced. E-CDC, enhanced CDC method; RPR, rapid plasma reagin.

Molecular typing of the *arp, tpr* and *tp0548* genes resulted in 99/136 (73%) fully typed samples, translating to 25 distinct E-CDC strain types (figure 1). The remaining 37 samples could only be partially typed. The *arp* genetic region was successfully amplified in 134/136 samples (99%), the *tpr* regions in 111/136 samples (82%) and *tp0548* region in 112/136 (82%) samples. Based on the *tp0548* region of the fully typed samples, 79/99 (80%) were SS14-like and 20/99 (20%) were Nichols-like. The most prevalent type was 14d/g, which was found in 23/99 samples (23%), followed by 14h/g (11%), 14k/g (11%) and 14d/f (9%). Most strain types (14/25, 56%) were found to occur in up to two samples. The patient of whom two ulcer swab samples at the same visit were included differed in *tpr* subtype resulting in types 14k/g and 14d/g.

Macrolide resistance among the different E-CDC strain types is shown in figure 1A. The 23S rDNA locus was successfully sequenced in 93/136 (68%) samples. The A2058G mutation was found in 83/93 (88%). No A2059G mutation was found.

The HIV status per E-CDC type (figure 1B) and the RPR titer per E-CDC type (figure 1C) showed no association with a particular TPA type. In addition, no significant association was found when comparing typable and untypable samples based on RPR titer and HIV status (see online supplementary table 1).

10.1136/sextrans-2019-054044.supp1 Supplementary data

## Discussion

A broad strain distribution was found. Few subtypes were clonally expanded, and most other subtypes were rare. This may be related to the wide variety of nationalities residing in Amsterdam. Most syphilis infections occur among MSM in the Netherlands.^1^ Sexual behaviour in the last 6 months was known for patients attending the STI clinic in Amsterdam, and 93% (74/80) of those with syphilis indeed reported being MSM. This percentage is similar to the percentage of reported MSM syphilis cases in STI clinics in the Netherlands in 2017 (93,5% of 1228 cases), confirming that we studied a representative population at risk for a syphilis infection.

A nested PCR was designed for the *arp* genetic region to increase the quality of the amplified product. The most prevalent strain type in this study was 14d/g, which is also found all over the world.^3 6^ Of the 99 fully typed samples 20% belonged to the Nichols-clade, which is relatively high compared with similar studies in Europe.^7 8^ A high diversity of TPA types was found in a rather homogeneous setting.

Only 93/136 samples (68%) were successfully sequenced for the 23S rDNA locus. Use of nested PCR for the amplification of the 23S rDNA locus may improve the relatively low rate of successfully sequenced samples. Most samples (83/93, 88%) contained the macrolide-associated mutation, A2058G, in agreement with other recent studies.^6^ Our results further discourage the use of macrolides for the treatment of syphilis. The A2058G mutation was not related to a specific strain type. As suggested by Arora *et al*,^8^ it is likely that extensive use of macrolides for treatment of diverse bacterial infections has played a significant role in emergence and spread of macrolide resistance in TPA.

It was hypothesised that the bacterial TPA load would be higher in samples derived from HIV infected syphilis patients due to immunodeficiency. In addition, a high RPR titer reflects a high immune response, possibly resulting in a lower bacterial load. Therefore, both RPR and HIV status might have been associated with typability of the samples. However, no association was found in our study. A possible limitation of this study is the absence of TPA DNA bacterial load based on qPCR.

The E-CDC typing method is time consuming and sometimes unreliable especially for *arp* and *tpr* genetic analyses. In this study, the two samples that derived from one patient differed in their *tpr* genetic region. This discrepant finding further enforces previous work suggesting genetic instability for this region.^9^ Future molecular TPA typing studies could be improved by using whole genome sequencing (WGS) with a higher resolution.^8^ Culturing of TPA is possible nowadays,^10^ which would facilitate WGS of TPA strains. However, culturing direct patient samples in a routine procedure has not yet been described. A robust and reliable method is multilocus sequence typing, which is possibly best suited for typing purposes.^7^

In conclusion, a broad strain distribution was found. Few subtypes were clonally expanded, and most other subtypes were rare. No associations were found between our typing results and the clinical and demographic data. In addition, 88% macrolide resistance shows the importance of discouraging macrolide use to treat syphilis.
