# Supplementary material for: Molecular diversity of Treponema pallidum subspecies pallidum isolates in Amsterdam, the Netherlands
Source: Sex Transm Infect. 2019 Aug 5;96(3):223–6. doi: 10.1136/sextrans-2019-054044 (PMC7231448; doi:10.1136/sextrans-2019-054044)
Supplement: Supplementary data [file sextrans-2019-054044supp001.pdf]

Supplementary data

Supplementary table 1

Factor		Not (fully) typed (%)	Fully typed (%)	p-value
HIV status	HIV positive	9 (31)	20 (69)	0,395
	HIV negative	11 (22,4)	38 (77,6)	
	Unknown	1 (50)	1 (50)	
RPR titer	Negative	4 (25)	13 (76,5)	0,797
	Low (1:1 to 1:2)	3 (18,8)	13 (81,2)	
	Middle (1:4 to 1:16)	10 (32,3)	21 (67,7)	
	High (1:32 to 1:258)	4 (25)	12 (75)	

Supplementary Table 1: Cross tabulation of demographic and clinical factors from 80 patients visiting the STI clinic regarding the ability to obtain a full E-CDC TPA type comparing the typable and untypable isolates. Included factors are HIV status and RPR titer. Fisher's Exact Test was used and a significant association was found if $p<0.05$.
